# PCD Genes—From Patients to Model Organisms and Back to Humans

**DOI:** 10.3390/ijms23031749

**Published:** 2022-02-03

**Authors:** Michal Niziolek, Marta Bicka, Anna Osinka, Zuzanna Samsel, Justyna Sekretarska, Martyna Poprzeczko, Rafal Bazan, Hanna Fabczak, Ewa Joachimiak, Dorota Wloga

**Affiliations:** 1 Laboratory of Cytoskeleton and Cilia Biology, Nencki Institute of Experimental Biology, Polish Academy of Sciences, 3 Pasteur Street, 02-093 Warsaw, Poland; m.niziolek@nencki.edu.pl (M.N.); m.bicka@nencki.edu.pl (M.B.); a.osinka@nencki.edu.pl (A.O.); z.samsel@nencki.edu.pl (Z.S.); j.sekretarska@nencki.edu.pl (J.S.); martyna.poprzeczko@wum.edu.pl (M.P.); raflik@gmail.com (R.B.); h.fabczak@nencki.edu.pl (H.F.); 2 Faculty of Chemistry, University of Warsaw, 1 Pasteur Street, 02-093 Warsaw, Poland; 3 Laboratory of Immunology, Mossakowski Medical Research Institute, Polish Academy of Sciences, 5 Pawinskiego Street, 02-106 Warsaw, Poland

**Keywords:** primary ciliary dyskinesia, mice, zebrafish, *Xenopus*, planarian, *Trypanosoma*, *Chlamydomonas*, *Paramecium*, *Tetrahymena*

## Abstract

Primary ciliary dyskinesia (PCD) is a hereditary genetic disorder caused by the lack of motile cilia or the assembxly of dysfunctional ones. This rare human disease affects 1 out of 10,000–20,000 individuals and is caused by mutations in at least 50 genes. The past twenty years brought significant progress in the identification of PCD-causative genes and in our understanding of the connections between causative mutations and ciliary defects observed in affected individuals. These scientific advances have been achieved, among others, due to the extensive motile cilia-related research conducted using several model organisms, ranging from protists to mammals. These are unicellular organisms such as the green alga *Chlamydomonas*, the parasitic protist *Trypanosoma*, and free-living ciliates, *Tetrahymena* and *Paramecium*, the invertebrate *Schmidtea*, and vertebrates such as zebrafish, *Xenopus*, and mouse. Establishing such evolutionarily distant experimental models with different levels of cell or body complexity was possible because both basic motile cilia ultrastructure and protein composition are highly conserved throughout evolution. Here, we characterize model organisms commonly used to study PCD-related genes, highlight their pros and cons, and summarize experimental data collected using these models.

## 1. Introduction—Cilia Diversity

A considerable number of human diseases are caused by genetic mutations that can be passed down from an individual carrying the mutation to the offspring. Ciliopathies are an example of such hereditary genetic disorders with a common feature—the assembly of dysfunctional cilia or their absence. 

Cilia, the hair-like cell protrusions play an essential role in human development, body functioning, and reproduction. Based on the ultrastructural organization and functions, cilia can be divided into two main categories: immotile sensory cilia and motile cilia that actively beat. In mammals, sensory cilia are most often formed as a single structure. The most ubiquitous are so-called primary cilia. These a few micrometers long protrusions are formed by the vast majority of non-dividing cells [1,2]. In photoreceptor cells (rods and cones), the sensory cilium is uniquely modified to accommodate a large amount of light-absorbing molecules [3,4]. In the inner ear, an immotile kinocilium assembled by cochlear hair cells is accompanied by numerous highly organized actin-containing mechanosensory protrusions called stereocilia or stereovilli and plays a role in their proper arrangement [5,6]. In contrast to these monociliated cells, olfactory sensory neurons are multiciliated and form up to 10–30 olfactory cilia (that are usually 50–60 µm long) on the dendritic knob [5,7]. 

Generally, motile cilia arise as multiple organelles per cell (from several dozen to ~250) [8]. These 5–10 µm long organelles are assembled by epithelial cells lining nasal passages, respiratory tracts [9,10,11], brain ventricles (in mouse, up to 50 cilia in E1 ependymal cells but one or two cilia in E2 cells (of note, E3 cells assemble single short primary cilium)) [12,13,14,15], oviducts [16,17], and efferent ducts connecting testis with epididymis [18,19,20,21,22,23]. Unusual motile cilia are assembled as monocilia by sperm cells (called flagella) and cells in the embryonic node [24,25]. The sperm’s flagella are significantly longer (~60 µm) than cilia assembled by the epithelial cells and contain additional sperm flagella-specific structures, while nodal cilia lack some of the typical motile cilia components [25,26,27,28]. 

Cilia of all types contain a uniquely organized microtubular scaffold (an axoneme) that originates from the basal body, which serves as the axoneme template and anchors cilium to the cell surface. A short fragment immediately distal to the basal body is called a transition zone and contains peripheral doublets and unique multiprotein complexes (Y-links) but lacks central microtubules (which originate above this region). The transition zone, together with transition fibers, functions as a ciliary gate [29,30]. 

In most sensory cilia, the axoneme (at least in the proximal part of the cilium) is composed of nine peripherally positioned microtubular doublets (9 × 2 + 0 organization) that are extensions of two more inner tubules of basal body triplets (a peripheral doublet is composed of a complete 13-protofilament tubule A and 11-protofilament tubule B attached to the wall of tubule A and thus sharing some of tubule A protofilaments; worth mentioning, out of these 11 protofilaments, only 10 are built of tubulin heterodimers). Interestingly, it was recently shown that in primary cilia from MDCK-II cells, the ciliary microtubules are accompanied by actin filaments [31].

**Figure 1:**
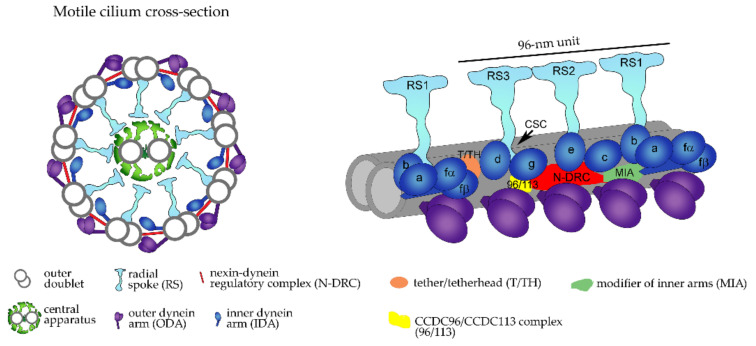
Schematic representation of the motile cilium ultrastructure. Cilium cross-section (on the left, view from the side of the basal body) and a 96 nm-long fragment of the outer doublet with docked ciliary complexes (called the 96 nm axonemal unit, on the right). Each axonemal unit contains four outer dynein arms (ODAs, in violet); seven inner dynein arms: heterodimeric (α, β) IDAf/I1 and single-headed IDA a, b, c, e, g, and d, (dark blue); the nexin–dynein regulatory complex (N-DRC, red) that coordinates the activity of ciliary complexes within the axonemal unit and connects two adjacent outer doublets; and three radial spokes (RS, cyan) that transiently interact with central apparatus projections via their heads, while the base of the stalk comes in contact with two different IDAs [32]. The main complexes are accompanied by several minor complexes that modulate and/or connect large ciliary complexes (e.g., tether/tether head complex (T/TH, orange [33]) positioned near IDA f/I1, CSC complex positioned at the bases of RS2 and RS3 (location indicated by an arrow) [34,35], MIA complex (green) connecting N-DRC and IDA f/I1 [36], and CCDC113/CCDC96 linker (yellow) connecting RS3, N-DRC, and IDAg [37].

Typical motile cilia and sperm flagella, besides peripheral doublets, also have two centrally positioned singlet microtubules (9 × 2 + 2 organization). Moreover, in typical motile cilia and sperm flagella, both peripheral and central microtubules serve as docking sites for numerous multiprotein complexes, forming a highly organized pattern along the entire cilium length (Figure 1).

Outer and inner dynein arms (ODAs and IDAs), radial spokes (RSs), and nexin links (N-DRCs) are the main complexes attached to peripheral doublets, while projections and a connecting bridge are central microtubule-associated structures [32,38]. 

In some cell types, along the cilium length, the microtubules’ configuration diverges from the most common patterns of organization. Recent studies on primary cilia revealed that 9 × 2 + 0 organization is apparent in the proximal part of the cilia, while in the distal part, microtubules form a bundle of gradually terminating doublets and singlets (A tubules) [31,39]. The number of ciliary microtubules also undergoes a reduction in the distal part of the photoreceptors and olfactory cilia. Photoreceptors can be morphologically and functionally divided into three compartments: (i) the most distal, light-sensitive outer segment, (ii) the organelle-containing (including cilium basal body) inner segment, and (iii) the connecting cilium, enabling transport between the inner and outer segments. In the connecting cilium, microtubules have a 9 × 2 + 0 organization, while in the outer segment, doublets are gradually reduced to singlets, and microtubules dislocate and lose the nine-fold symmetry [4,5]. Similarly, olfactory cilia have the 9 × 2 + 2 configuration in the proximal cilium, but peripheral doublets are reduced to singlets in the distal part (worthy of note is that these cilia are immotile as they lack dynein arms) [5,9]. Ultrastructural studies on mice and guinea pigs showed that the sensory kinocilium in the auditory organ has a 9 × 2 + 2 organization [5,40]. However, some researchers have also observed cilia missing the central apparatus (9 × 2 + 0) [41,42]. Kinocilia have some of the motile cilia multiprotein complexes such as the outer dynein arms and radial spokes but lack the inner dynein arms [6,42]. 

The sensory cilia function as antennas that receive signals from the surrounding environment and transduce them to the cell body. Although motile cilia can also perform sensory functions [43,44], their primary role is to generate a shift or circulation of the surrounding environment, or to enable cell motility. In humans, the coordinated movement of motile cilia enables the expulsion of the inhaled bacteria and particles, together with the mucus produced by the epithelial cells, out of the respiratory system (mucociliary clearance), generates a circulation of the cerebrospinal fluid through the brain ventricles, supports the transport of the oocyte and early embryo in the Fallopian tube, and likely, as in mouse, the passage of sperm cells through efferent ducts [16,23,45,46,47,48]. Long, single flagellum enables sperm cell motility. During embryonic development, the rotatory movement of nodal cilia generates the left-directed nodal flow and consequently plays a role in establishing the left–right asymmetry of the visceral body organs [28,49]. 

The lack or improper functioning of motile cilia causes reoccurring respiratory infections that lead to bronchiectasis and lung damage; in some affected individuals cilia/flagella dysfunction results also in subfertility or infertility, laterality defects (~50% of affected individuals), and rarely, hydrocephalus [50].

Given the fact that in humans cilia play an important role during embryo development and in physiological processes taking place in already formed tissues and organs, it is not surprising that their dysfunctions cause severe body defects known as ciliopathies. At the base of the etiology of the vast majority of ciliopathies are defects of the primary cilia [51,52], while primary ciliary dyskinesia (PCD) and multiple morphological abnormalities of sperm (MMAF) are ciliopathies caused by alterations in motile cilia and/or sperm flagella. Similar to other ciliopathies, PCD is a heterogeneous disorder (mostly autosomal recessive) that affects 1 out of 10,000–20,000 individuals [50,53,54]. Up to now, mutations leading to PCD have been detected in nearly 50 genes encoding structural or regulatory proteins that control gene expression, cilia assembly, and functioning [53,55,56]. However, in many cases, the causative mutation(s) remains unknown. 

The recent years have brought about significant progress, not only in PCD diagnostics but also in our understanding of the molecular mechanisms behind cilia dysfunction. The advances in our comprehension of the processes regulating cilia formation and motility would not be possible without the extensive cilia-oriented studies conducted in diverse and, compared to humans, simpler model organisms, ranging from protists to mammals. Establishing experimental models with different levels of cell/body complexity was possible because both basic motile cilia ultrastructure and protein composition are highly conserved throughout evolution (it is believed that motile cilia were assembled by the last common ancestor of all current eukaryotes, LECA [57,58,59]). 

More importantly, the genomes of model organisms are fully sequenced, and the methods enabling genome manipulation and the engineering of gene knockouts and knock-ins, mutagenesis, or protein tagging are well-developed. Thus, nowadays, genetic techniques allow researchers to engineer organisms with modifications that correspond to mutations identified in PCD or MMAF-affected individuals and to analyze their outcome at the molecular, ultrastructural, and whole-organism levels. On the other hand, the basic research conducted in model organisms leads to the identification of new ciliary proteins and to the elucidation of their role in ciliogenesis and the regulation of cilia beating. As a result, knowledge regarding ciliary genes, including PCD causative genes, can be transferred in both directions—from patients to model organisms and from model organisms to humans. 

Before starting the PCD-related research using model organisms, it is important to ask a simple question: which model will be the most suitable to conduct the planned experiments? In this review, we briefly characterize model organisms commonly used to study the outcome of the mutation(s) in PCD-related genes, highlighting their pros and cons, and then summarize the collected experimental data showing the relationship between gene mutation and its phenotypic outcome in humans and model organisms.

## 2. Single-Celled Models—The Power of Small and Simple

In the case of a multicellular organism, mutations in ciliary genes can affect not only the function of the ciliated cell per se but also the entire development of the organ and/or body as many proteins are required for the assembly or activity of both sensory and motile cilia [45,60,61,62]. Moreover, some ciliary proteins may also play a role in non-cilia-related processes [63]. In consequence, mutations in such genes cause complex body defects or even death. Single-celled models facilitate the analyses of the outcome of mutations in ciliary genes solely on the phenotype of the cell carrying the mutation. Changes in the phenotype of unicellular model organisms that manifest by altered swimming, cilia length, or motion (ciliary waveform, amplitude, and frequency) can be easily monitored using phase-contrast microscopy or dark-field microscopy combined with a high-speed camera as cilia are directly accessible. Furthermore, established microscopic techniques (transmission electron microscopy (TEM) and cryo-electron tomography (cryo-ET)) combined with methods of genome manipulation enable not only the analysis of cilia ultrastructural defects but also the precise localization of newly identified ciliary proteins. Finally, the maintenance of single-celled models is relatively easy and inexpensive, and a short generation time ensures the availability of a large volume of cultures and the purification of a sufficient amount of ciliary proteins for large-scale proteomic and biochemical analyses. 

Equally important is the fact that the use of single-celled models is in agreement with the 3Rs principle—Replacement, Reduction, and Refinement—which was developed to minimize the number of consciously-living higher animals used in scientific experiments [64].

On the other hand, unicellular models and humans are evolutionarily distant species, and while some ciliary genes are highly conserved, other PCD-causative genes are less conserved or not present in the unicellular models’ genomes. Moreover, many ciliary proteins are specific only to unicellular models, as was shown by the proteomic analyses of total ciliomes [65,66,67].

### 2.1. Green Alga, Chlamydomonas reinhardtii

*Chlamydomonas reinhardtii* is undoubtedly a splendid model organism for the study of many aspects of motile cilia/flagella assembly and functioning, and without a doubt, for a long time, *Chlamydomonas* was a leading model used by cell biologists to discover the identity, precise intraciliary localization, and the role of ciliary proteins. Subsequently, a lot of the genes encoding these proteins turned out to be the PCD-causative genes, and the knowledge gained during studies on green alga helped to understand the ultrastructural changes behind the cilia dysfunction in affected individuals, especially since the cryo-ET era began.

On the other hand, although the cilia ultrastructure, in general, is highly evolutionarily conserved, in *Chlamydomonas* flagella, in contrast to humans and other model organisms, only two radial spokes are full-length structures (RS1 and RS2), while RS3 is reduced to the short knob. Such truncation excludes direct interactions between RS3 and a central apparatus [68,69]. Thus, some differences in the regulation of *Chlamydomonas* flagella and human cilia beating cannot be excluded. 

**Figure 2:**
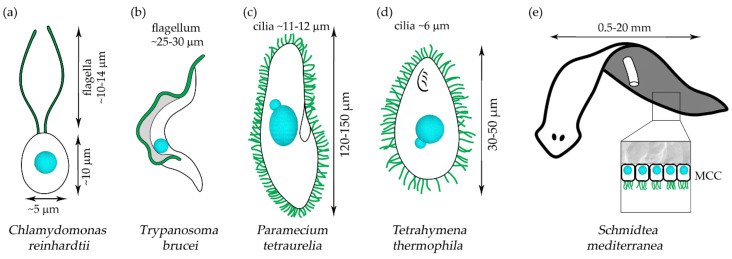
A schematic representation of the non–vertebrate PCD model organisms, the unicellular (**a**) Green alga *Chlamydomonas reinhardtii*; (**b**) Parasitic protist, *Trypanosoma brucei* (bloodstream-form) and two free–living ciliates, (**c**) *Paramecium tetraurelia* and (**d**) *Tetrahymena thermophila* as well as the multicellular (**e**) Freshwater planarian flatworm, *Schmidtea mediterranea,* with an enlarged fragment showing the multiciliated cells (MCCs) of the ventral epidermis. Nucleus (cyan), motile 9 × 2 + 2 cilia/flagella (green).

This small, oval-shaped green alga assembles two motile flagella that are longer (~10–14 µm) than the cell itself (Figure 2a) [70,71]. In the laboratory, *Chlamydomonas* can be grown in both liquid and solid media, and under optimal conditions and constant light it can multiply every 8–10 h [71,72]. Such a short generation time ensures that a large number of cells, and consequently a large amount of ciliary proteins, can be obtained in a relatively short time. 

*Chlamydomonas* cells are haploid; hence, mutations are immediately expressed, affecting cell phenotype and in the case of ciliary genes—flagella assembly and/or motion (worthy of note is the fact that old flagella are retracted before division, and new flagella are assembled based on the expression from a modified genome). *Chlamydomonas* mutants can be generated by exposure to UV radiation or chemical agents, or by insertional mutagenesis (random integration of DNA fragments containing a selectable marker (non-homologous recombination), causing gene inactivation). Molecular mapping followed by rescue experiments or whole-genome sequencing enables the identification of mutated loci [73,74]. More importantly, mutations in different loci can be combined and expressed in a single cell by mating cells carrying particular mutations [75]. Conveniently, the libraries of *Chlamydomonas* insertional mutants are commercially available [76,77].

Not only forward genetics but also reverse genetic tools are well established for *Chlamydomonas,* and enable both the expression of recombinant proteins and gene silencing [73,74]. Genome editing, including knockout and knock-in, using the CRISPR/Cas9 approach (clustered regularly interspaced short palindromic repeats and CRISPR-associated protein 9) was also adopted [78,79,80], but as of yet, it is not broadly used. Recently, a modified, more efficient version known as TIM (targeted insertional mutagenesis) was proposed [81]. 

The DNA fragments can be delivered to the cell by several methods, including biolistic transformation, DNA-coated glass beads, and the most efficient and broadly used method—electroporation [75,82,83,84,85,86,87].

The well-established cell biology methods enable not only the analysis of ultrastructural changes in the cilia but also an investigation into how these structural changes translate into alterations in flagella movement and cell motility (from mild defects to cell paralysis) [88]. For example, *CCDC39/FAP59* and *CCDC40/FAP172*, whose mutations (next to mutations in genes that encode dynein arms) are frequent causes of PCD, were shown to form a filament that functions as a molecular ruler, marking not only the length of the 96 nm axonemal repeat but also a position of the main ciliary complexes such as IDAs, N-DRC, and RSs, but not ODAs [89]. The positioning/attachment of ODAs is affected by a product of *CCDC103*, another PCD-causative gene, as was also discovered in *Chlamydomonas* [90,91]. Studies conducted in green alga also revealed that PCD-related DRC1, 2, and 4 proteins form a core of the N-DRC, and that large parts of N-DRC are missing when genes encoding these proteins are mutated [92]. N-DRC is a main ciliary hub that coordinates and regulates other ciliary complexes of the 96-axonemal unit and connects adjacent outer doublets [92]. Using *Chlamydomonas* as a model, it was also shown for the first time that the *RSP3* gene encodes a component of the RS stem, and that the *RSP3* mutants lack the entire RS structure while the RSP1, 4, and 9 proteins build the RS head, and their lack affects only this part of the RS complex [68,69,93,94,95]. *FAP57/WDR65* is another PCD-causative gene [96], and the encoded protein forms a filament-like structure extending along the 96 nm axonemal unit, as was shown in *Chlamydomonas* [97]. Finally, *CPC1/SPEF2* [98], *FAP221/PCDP1* [99], *Hydin* [100], and *PF16/SPAG6* [101,102] (recently revealed to cause PCD [103]) were shown to encode components of the central apparatus structure, and mutations in these genes caused the lack of C1b-C1f, C1d, C2b-C2c, or C1a-c-e projections, respectively. 

Besides early studies showing the significance of the structural components of dynein arms for flagella motility—such as dynein heavy chain, *oda2* [104], the two intermediate chains *IC78/IC1* and *IC69/IC2* [105,106] (orthologs of the human PCD-related genes *DNAH5*, *DNAI1*, and *DNAI2* [107,108,109,110]) —*Chlamydomonas* was used as a model to verify the importance of genes that have recently been found to be mutated in patients having cilia without dynein arms. These genes encode cytoplasmic proteins required for dynein arm assembly: CFAP298/FBB18 [111], CFAP300/FBB5 [112], DNAAF1/LRRC50/ODA7 [113,114,115], DNAAF2/KTU/PF13 [114,116,117,118], DNAAF3/PF22 [114,116,119], DNAAF4/PF23/DYX1C1 [116,120], HEATR2 [121], MOT48/IDA10 [118,122]; or for transport—LRRC56/ODA8 [123] (for review, see [124]). 

Interestingly, in some patients with detected fertility and laterality defects, the respiratory problems were not reported or were mild and thus did not fulfill the PCD criteria (e.g., in individuals with mutations in the *CCDC19/CFAP45* and *WDR16/CFAP52* [22]). The basic studies conducted in *Chlamydomonas,* among others, revealed that proteins encoded by such genes are not components of the main ciliary complexes, and that their lack does not affect or mildly affects flagella motion (reduces beating frequency and/or amplitude and waveform). For example, the abovementioned CFAP45 and CFAP52 are microtubule-binding proteins attached to the inner (luminal) wall of the outer doublet B-tubule and are likely stabilizing outer doublets [125]. Similarly, several genes recently shown to be MMAF-causative genes were identified as encoding proteins positioned near the radial spoke base or dynein arms. These are FAP61, FAP91, and FAP251 forming the CSC complex [34,35], the RS2–base protein, FAP206 [126], and FAP43 and FAP44, forming the tether/tetherhead complex positioned near the motor domains of the heterodimeric IDAf/I1 and regulating IDAf/I1 activity [127]. 

### 2.2. Parasitic Protists, Trypanosoma spp. 

Trypanosomes are broadly studied, uniflagellated, parasitic protists that cause tropical diseases such as sleeping sickness (*T. brucei*) or Chagas disease (*T. cruzi*). Taking advantage of the collected general knowledge on the culture condition, genome modifications, and biology of Trypanosomes gained during the analyses of these parasites, researchers “converted” *Trypanosoma* spp. into a model to study PCD. *T. brucei* has a two-host multi-stage life cycle, and cells at different developmental stages vary in their size and flagellum length. In the so-called short epimastigote, which is the smallest of all *T. brucei* forms, the flagellum is only ~3 µm long. In metacyclic trypomastigotes and attached epimastigotes, the flagellum length ranges from 13 to 16 µm, while the procyclic form has ~20 µm flagella. The longest flagella (~25–30 µm) are assembled by the bloodstream slender form, long trypomastigotes, and long epimastigotes [128,129].

The flagellum (except for short epimastigote) is docked in the cell posterior end and extends parallel to the cell surface, in the direction of the cell anterior end and beyond it (Figure 2b). Most of the flagellum (more precisely, the flagellar membrane) is laterally connected to the cell by a membranous-cytoskeletal structure called a flagellar attachment zone (FAZ) formed by both flagellum and cell surface. The most distal, unattached part of the flagellum is short and comprises roughly one-tenth up to one-seventh of the entire flagellum length (at least in epimastigote-like cells) [130]. Besides the axoneme composed of typical for motile cilia components, Trypanosomes’ flagellum contains an additional, unique structure called the paraflagellar rod, extending along the axoneme length parallel to doublets 4–7 [131]. 

During cell division, old and newly assembled flagella co-exist until the daughter cells separate. Thus, in cells with knocked down ciliary genes, such a phenomenon provides an opportunity for comparing the ultrastructure and behavior of old unaffected flagellum and new flagellum formed after the elimination of the protein of interest. Hence, differences in the ultrastructure and motility between old and new flagella can be specifically attributed to the function of the targeted protein. 

The procyclic stage (present in tsetse fly midgut) and bloodstream form of *Trypanosoma brucei* can be cultured in vitro, in both liquid and solid media. The average doubling time of the strain commonly used in the laboratory is approximately 6–8 h for the slender bloodstream form and 8–9 h for the procyclic form [132]. More importantly, both forms can be stored deep-frozen for a long time. 

The available molecular tools enable the stable or transient expression of tagged proteins under the control of a native promoter or their overexpression, as well as gene silencing using a tetracycline-inducible RNAi system [132,133,134]. The DNA constructs can be introduced into the cell by the highly effective electroporation or by nucleofection [132,135,136,137] and efficiently incorporated into the genome via homologous recombination [132]. The recent adaptation of the CRISPR/Cas9 method expanded the repertoire of *Trypanosoma* genome manipulation [137,138,139,140]. The effect of the mutation or knockout of ciliary genes on cilia assembly or motion can be evaluated by the direct observation of the flagella movement and the analysis of the pattern of cell swimming using video microscopy, or less precisely, by the sedimentation assay [132,141,142,143,144]. At the ultrastructural level, not only classical SEM and TEM but also cryo-ET approaches were successfully applied [145,146,147].

Although to a lesser extent than *Chlamydomonas*, *Trypanosoma* was also used as a model in both basic studies aiming to discover the role of ciliary proteins (including those that were later shown to be implicated in PCD etiology) and in verifying that genes identified as causative in PCD-affected individuals are indeed responsible for cilia/flagella motility alterations. For example, the knockdown of the component of motile flagella 70, *CMF70*, and *trypanin*, orthologs of DRC2 and DRC4, respectively, resulted in uncoordinated flagella beating that hardly translated into cell movement (similar as in patients with *DRC2* or *DRC4* mutations) [148,149]. The knockdown of *hydin* also significantly affected *Trypanosoma* cells’ motility and caused ultrastructural changes such as the rotation of the central apparatus and the lack of one (7%) or both (19%) central microtubules [150]. Some flagella without one (5–12%) or both (3–5%) central microtubules were also observed in *Chlamydomonas hydin* mutants; however, the majority of *Chlamydomonas* flagella lacked C2b and a part of the C2c central apparatus projections [100]. It is likely that in *Trypanosoma* mutant flagella, the C2b projection was also missing, but structural defects were probably too subtle and thus not apparent in TEM images. In contrast, the knockdown of PF16/SPAG6 in *Trypanosoma*, another central apparatus protein, only led to the rotation of the central apparatus [142].

In *Trypanosoma*, as in other organisms, the knockdown of *RSP3* [142] affected radial spoke assembly and cell motility, while the formation of dynein arms was reduced in cells with knocked out genes encoding the dynein arm structural protein, DNAI1 [143], dynein arm assembly factors (DNAAF1/LRRC50/ODA7) [113], or LRRC56/ODA8, a protein required for the transport of dynein arms to the distal part of the axoneme [151]. On the other hand, mutants with the knockdown of *TbLRTP* encoding a protein with some similarity to LRRC6/DNAAF11 as suggested by [152] exhibited a different phenotype; while cilia in PCD-affected individuals carrying *LRRC6* mutation lack ODAs and IDAs [153], flagella of *Trypanosoma* cells with a reduced level of TbLRTP were described as having a normal ultrastructure (the presence or lack of dynein arms was not specifically addressed) [154]. Moreover, the cell proliferation rate was reduced in the TbLRTP mutant, thus casting doubt as to whether this is a true functional homolog of mammalian LRRC6. 

*Trypanosoma* cells were also used as a model to elucidate the flagellar role of proteins encoded by the MMAF-causative genes. These were CFAP43 and CFAP44 [155], CFAP91 [156], CFAP251/WDR66 [157], and TTC29 [158]. Surprisingly, CFAP43 and CFAP44, which were shown to build small tether/tetherhead complex in *Chlamydomonas* and *Tetrahymena* [127], were found to be located between the paraflagellar rod and outer doublets 5 and 6 in *Trypanosoma* [155]. However, the localization of CFAP43 and CFAP44 in *Trypanosoma* was addressed using STED super-resolution microscopy, while the visualization of the tether/tetherhead complex in cilia and flagella of free-living model organisms was performed using cryo-ET. Thus, one cannot exclude that CFAP43 and CFAP44 have dual localization in *Trypanosoma*, near IDA f/I1 as in other ciliated organisms, and in *Trypanosoma*-specific organisms, between the paraflagellar rod and axonemal outer-doublets.

### 2.3. Ciliate: Tetrahymena and Paramecium

In contrast to *Trypanosoma* assembling a single flagellum or bi-flagellated *Chlamydomonas*, free-living, fresh-water organisms that belong to ciliates, *Tetrahymena* and *Paramecium* (Figure 2c,d), have several hundred cilia that enable cell locomotion (somatic cilia) or direct food particles into the oral cavity (oral cilia). *Tetrahymena thermophila* and two species of *Paramecium*, *P.tetraurelia*, and *P.caudatum,* are well-established laboratory models. In the natural environment, they feed on bacteria, but in the laboratory, these species can be maintained on axenic media. An interesting feature of ciliates is the presence of two types of nuclei, the generally transcriptionally silent diploid micronucleus and the transcriptionally active ampliploid macronucleus (not the entire genome is amplified) [159]. 

*Tetrahymena* is 30–50 µm long and forms ~600 cilia with the average length of ~6 µm. *Paramecium* is a larger ciliate (*P.tetraurelia* is 120–150 µm long, while *P.caudatum* is ~300 µm) and assembles ~4000–5000 cilia that are ~10–12 µm long [160,161,162,163,164]. When grown under optimal conditions, *Tetrahymena* divides every 2.5–3 h, while *Paramecium* requires twice as much time (*P.caudatum*, 7.6–8.4 h [165], *P.tetraurelia*, 5–14 h (depending upon culture conditions, including medium type) [166]). Short generation time and low-cost culture conditions, together with established methods of triggered cilia shedding followed by their fast (~2 h) and synchronous regeneration, enable the large-scale biochemical studies of proteins in assembling and full-length cilia. Furthermore, the microscopic techniques enabling analyses at the cellular and ultrastructural levels are well-developed [167,168,169,170,171,172]. 

In the case of *Tetrahymena*, not only microscopic methods but also biochemical approaches and tools for reverse and forward genetics are well-developed. Appropriate constructs that enable gene deletion, mutagenesis, or protein tagging for localization studies (GFP, HA, V5) or biochemical experiments (BioID) can be introduced to the cell using biolistic transformation and be incorporated into the micro- or macronuclear genome via homologous recombination. The expression of fusion proteins can be controlled either by a native promoter or a cadmium-inducible metallothionein promoter, leading to protein overexpression [173,174,175,176]. 

While changes in the macronuclear genome affect cell phenotype, mutations in the micronucleus are “silent” and manifest after a sexual process (conjugation) when new macronuclei are formed based on the micronuclear genome of two conjugating cells. Such nuclear dualism is advantageous while investigating the role of essential genes as lethal mutations can be “stored” in micronuclei (and thus a mutated strain can be maintained) and expressed after conjugation [174,177]. 

Because ciliates use cilia for cellular motility and feeding, defects in cilia affect cells’ swimming phenotype (cell velocity, shape of swimming trajectories, ciliary beating pattern, amplitude, and/or frequency) and food particle uptake (phagocytosis). Both these processes can be monitored using light microscopy [167].

In comparison to *Tetrahymena*, there are fewer approaches enabling genome manipulation in *Paramecium*. For localization studies, constructs enabling the expression of GFP-tagged proteins are introduced to the macronucleus by microinjection. To silence gene expression (RNAi-based knockdown), *Paramecium* is usually fed with bacteria, but the microinjection of dsRNA is also possible [163,178,179]. Another challenge is *Paramecium* cells’ senescence and long-term storage. 

Recently, it was demonstrated that the knockdown of ciliary genes in *Paramecium* causes defects similar to those observed in PCD-affected individuals. The deletion of the ortholog of human DNAH9, a dynein heavy chain that, together with DNAH5, are components of the ODAs in the distal part of the respiratory cilia [180,181], reduces the number of ODAs in *Paramecium* cilia [182]. Furthermore, cilia in *Paramecium* cells with the silenced expression of *ZMYND10* (Zinc finger MYND-type containing 10) [183], *TTC12* (Tetratricopeptide repeat protein 12) [184], or *CFAP300/FBB5/C11orf70* [112] had a reduced number of outer and inner dynein arms. More importantly, individuals with PCD caused by mutations in *ZMYND10* [185,186,187] or *CFAP300* [112,188] showed similar ultrastructural defects in respiratory cilia, while mutations in *TTC12* resulted only in the lack of IDAs [184]. Interestingly, in the same individuals carrying *TTC12* mutations, sperm flagella lacked both IDAs and ODAs [184]. The localization studies of the GFP-tagged CFAP300, TTC12, and ZMYND10 proteins showed that they are generally cytoplasmic, and only limited amounts are present in cilia [182,183,184]. These observations are in agreement with data obtained in other models, showing that investigated proteins are involved in dynein arm assembly [189].

Although *Tetrahymena* has not yet been used as a model to investigate newly identified PCD-related genes, research conducted on *Tetrahymena* shed some light on the structure and protein composition of different ciliary complexes required for cilia assembly and cilia beating regulation. The knockout of *SPEF2A*, a PCD-related gene encoding a subunit of the central apparatus that is essential for the assembly/stability of the entire C1b projection, changes the pattern of cilia beating to one that is rotatory-like [172]. A similar effect was caused by the deletion of *CFAP69* encoding another C1b protein [172]. Interestingly, in humans, mutations in genes orthologous to *SPEF2A* (*SPEF2*) and *CFAP69* turned out to cause MMAF and very rarely PCD (until now, only mutations in *SPEF2* were connected to PCD) [190,191,192,193,194,195]. Among other MMAF-causative genes [196,197] studied in *Tetrahymena* are, as mentioned before, *CFAP43* and *CFAP44* [127,198,199,200,201,202], the CSC subunits *CFAP61* [203,204] and *CFAP251* [204,205], and *CFAP206,* which forms a part of the RS2 base [126,196].

The presented examples clearly show how the detailed analyses at the molecular, ultrastructural, and proteomic levels conducted in single-celled models help to understand the cilia-related changes observed in PCD patients. 

## 3. Freshwater Planarian *Schmidtea mediterranea*—Matters Becoming a Little More Complicated

Although single-celled organisms are convenient models for the exploration of various aspects of cilia biology, certain questions regarding cilia assembly, beating regulation, and coordination cannot be addressed. A free-living planarian, *Schmidtea mediterranea,* used for over 100 years to investigate the regeneration process [206], has recently emerged as a model for the study of de novo centriole formation, planar cell polarity as well as motile cilia assembly and functioning [188,207,208,209,210]. As the name of the phylum suggests (flatworms, Platyhelminthes), the body of *Schmidtea* is flat and thin (Figure 2e). The adult form is ~20 mm long (the asexual strain is slightly smaller); however, under starvation conditions, the body size can be reduced to ~0.5 mm. The epithelial multiciliated cells (MCCs) of the ventral epidermis assemble classical motile cilia (~80 per cell) that mediate animal locomotion [207,211]. MCCs are also present in the epithelium lining a pharynx, the planarian feeding organ [208], and in protonephridia, the planarian excretory system where they are called “flame cells” [212,213]. Additionally, in auricles, structures participating in both chemo- and mechano-reception among ciliated epithelial cells are sensory neurons, assembling cilia with classical 9 × 2 + 2 microtubule organization, (however, it is unknown if those cilia are motile [214]). Because planarian MCCs share many features with vertebrate MCCs, data regarding basal body biogenesis and ciliogenesis obtained using *Schmidtea* as a model can shed a light on these processes in MCCs present in epithelia lining airways, brain ventricles, or oviduct in humans [215].

Although the repertoire of methods, especially those enabling genome manipulations in *Schmidtea,* is limited in comparison to those available for single-celled models, the planarian offers several advantages. Compared to vertebrate models, *Schmidtea* culture is simple and inexpensive as planarians can be maintained at 18–20 °C in the dark, in plastic, food-grade containers with an inorganic medium composed of common salts, and then fed once a week with calf liver homogenate. Before the experiment, a larger number of planarians can be obtained either by animal fission or by manual amputations, followed by the regeneration of obtained body fragments. Importantly, progenies obtained from a single animal, either by subsequent fissions or amputations, have the same genetic background, which is crucial while comparing control and experimental animals [216,217].

Methods enabling the expression of mutated or tagged proteins in *Schmidtea* are not yet available, but RNA interference (RNAi)-based gene knockdown is straightforward and efficient. RNAi can be introduced into planarian cells either by the microinjection of long double-stranded RNAs (dsRNA) or by feeding with an *E. coli* strain carrying a plasmid for dsRNA expression [211,218]. The amputation of the part of the planarian body ensures that MCCs in the regenerating fragment will lack or have a low level of expression of the targeted gene. The effect of the gene depletion can usually be observed in less than a week [211,218,219].

Similar to unicellular models, cilia assembled by planarian ventral epidermis and pharynx are easily accessible, and thus the effect of gene silencing on cilia assembly/functioning and subsequently planarian locomotion can be observed without difficulty. The ciliary waveform, beating frequency, and synchrony of cilia beating can be analyzed after recording the movement of cilia in the lateral part of the animal using a high-speed video camera and differential interference contrast (DIC) microscopy [219]. Changes in planarian locomotion can be described by measuring the speed of animal movement. The effect of gene silencing on cilia protein composition and ultrastructure can be revealed using the methods of immunofluorescence and electron microscopy, respectively [211,218,219].

Until now, only a limited number of proteins encoded by cilia-related genes has been studied using *Schmidtea* as a model. These are (i) FoxJ1-4, one of the key transcription factors controlling ciliogenesis in MCC cells [220], (ii) CFAP298/FBB18 [111] and CFAP300/FBB5/C11orf70 [188], the PCD-causative genes encoding cytoplasmic proteins that participate in dynein arm assembly/transport [112,188,221], (iii) DAW1/WDR69/ODA16 [209], an adaptor protein involved in outer dynein arm transport [222,223,224], not yet linked to PCD but likely a causative gene based on ultrastructural defects (reduced number of ODAs) in *Chlamydomonas* [225], zebrafish [226], and mouse [227] mutants, and (iv) CFAP45/CCDC19/NESG1 and CFAP52/WDR16 [22], two proteins that were shown to localize in the lumen of the outer doublet B-tubule in *Chlamydomonas* [125]. In humans, as mentioned above, mutations in either *CFAP45* or *CFAP52* cause mild respiratory distress, although without fulfilling the PCD diagnosis criteria. The affected individuals develop situs inversus and asthenospermia [22]. Importantly, in the case of all these proteins, their knockdown in *Schmidtea* affected cilia motility as in other model organisms. Thus, with the further development of tools for reverse genetics, it is likely that the planarian will become a valuable and popular PCD model. 

## 4. Aquatic Vertebrates–When a Plethora of Siblings and Fast Development Matter

As stated above, single-celled organisms and to less extent, a free-living flatworm, are convenient models to investigate the role of ciliary genes, including those related to PCD. However, certain motile cilia-related genes and processes (especially genes controlling embryo’s laterality or development and functioning of cilia-bearing organs) obviously cannot be studied in unicellular organisms or in planarian but require the employment of vertebrate models. 

**Figure 3:**
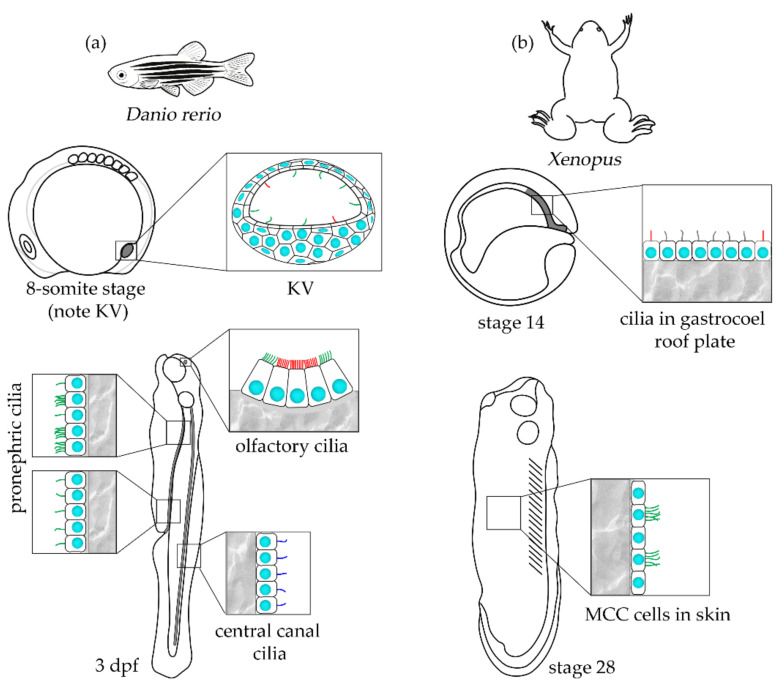
A schematic representation of the larval and adult forms of vertebrate PCD models, (**a**) zebrafish and (**b**) *Xenopus*. The adult animals reach the following sizes: zebrafish ~3.5 cm; *X. laevis,* males between 4.5 and 9.8 cm, females from 5.7 to 14.7 cm; *X. tropicalis,* males between 3.2 and 3.9 cm, females between 4.8 and 5.5 cm (https://zfin.org/zf_info/zfbook/stages/ accessed on 30 December 2021 [239], http://www.xenbase.org accessed on 30 December 2021 [240]). Nuclei (cyan), motile 9 × 2 + 2 cilia (green), motile 9 × 2 + 0 central canal cilia (navy blue), immotile cilia (red), motile cilia in gastrocoel roof plate of unknown microtubule configuration (grey).

Embryos of zebrafish (*Danio rerio*) and two species of *Xenopus*, the South African clawed frog, *X. laevis* and smaller Western clawed frog, *X. tropicalis,* are well-established models to study diverse biological processes, including those involving motile cilia (Figure 3). Both these aquatic vertebrates produce a huge number of oocytes that after external fertilization develop outside of the mother’s body, enabling direct observations of the outcome of cilia activity and defects caused by cilia dysfunction in a reliable number of animals. 

Zebrafish females reach sexual maturity after about three months and weekly can lay even 100–300 eggs (with a diameter ~0.7 mm) [228,229]. *Xenopus* species require at least twice that time to reach the sexual maturity—5.5–7.5 months post-metamorphosis (PM, a transition from a tadpole to adult form that usually takes place within 2–3 months after fertilization) in the case of *X. tropicalis* and 10–24 months PM in the case of *X. laevis* [230]. *Xenopus* females lay more than 1000 eggs per mating [231], and similar to zebrafish, oocytes are large (~1.3 mm in *X. laevis* and 0.8 mm in *X.tropicalis*) [232]. In contrast to diploid *X. tropicalis*, the genome of the *X. laevis* has undergone duplication and is hence tetraploid, making genetic manipulations difficult. Thus, larger embryos of the *X. laevis* are preferentially used in microscopic or transplantation studies, while genome modifications are performed in *X. tropicalis* embryos [233,234,235]. 

In both zebrafish and *Xenopus,* motile cilia appear early in the embryonic development. In zebrafish embryos, motile cilia are first formed in the lumen of a transient, fluid-filled structure homologous to mammalian node, the left-right organizer, LRO, called a Kupffer’s vesicle (KV). KV is present in embryos at 4–14 somite stages (~11–18 hpf, hours postfertilization) and is involved in establishing the left-right body organs’ asymmetry. Interestingly, not all KV’s cilia are motile. The number of motile cilia increases, and the pattern of fluid flow changes as KV’s development advances. Thus, cilia motion in the KV is usually studied in embryos at 6–10 somite stages [228,236,237,238]. Both motile and immotile cilia are assembled as a single structure per cell, and at 8-somite stage, their length has been estimated to be ~3.3 µm in fixed embryos [236] and from 4 to 12 µm (on average, 6–8 µm) when cilia were imaged in live or fixed embryos [237]. 

As zebrafish larvae further grow (approximately 24–72 hpf larvae are ~1.2–1.7 mm long), motile cilia are formed in the olfactory placode, pronephros, brain ventricles, and spinal central canal [236,241]. Cilia in the spinal canal lumen, similar to cilia in the KV, are assembled as a single structure per cell and exhibit rotational movement, but in contrast to the KV where the vast majority of cilia have 9 × 2 + 2 microtubule organization [237,242], cilia in the spinal canal show a 9 × 2 + 0 microtubule pattern [236]. Their rotational motion contributes to the cerebrospinal fluid flow [236]. Cilia in spinal central canal are ~2.1 µm [236].

Epithelial cells lining pronephric ducts can be both mono- and multiciliated, and cilia reach ~8.8 µm [236]. Mono- and multiciliated cells are present in the anterior and middle part of the pronephric ducts, while in the posterior part, cells are mostly monociliated [236,243,244]. It is worth pointing out that monocilia are formed earlier (~24 hpf) than cilia in multiciliated cells (~2 days) [244]. 

Cells of the olfactory placode assemble both, sensory and motile cilia. The latter ones are assembled as multicilia, mainly by cells at the olfactory placode periphery [242]. 

Zebrafish embryos with mutations in ciliary genes develop apparent phenotypic changes such as ventral body curvature, hydrocephalus, and pronephric swellings or formation of kidney cysts. A more detailed examination can also reveal abnormal otolith deposition and altered left-right body asymmetry, including random heart jogging and looping [245,246].

Similar as in zebrafish embryos, motile cilia in *Xenopus* embryos, are first formed as monocilia in the transient structure called the gastrocoel roof plate, playing the role of the left-right organizer [247,248]. LRO cilia start to assemble at stage 14 (~16 hpf) and at stage 16–18 (~18–20 hpf), approximately 250–270 cilia of ~5–6 µm beat rotationally, causing leftward fluid flow ([249,250], https://www.xenbase.org/anatomy/alldev.do accessed on 30 December 2021). Cells assembling motile cilia are bordered by cells forming non-motile cilia [247]. The effect of LRO cilia activity (position of developing heart and other organs) can be analyzed in 3–4 days old larvae [248,251]. 

In *Xenopus,* motile cilia formed by the larva and tadpole skin (stages 20–50, larvae hatch at the stages 35–36) are the most often studied cilia [252]. They are assembled as multiple, polarized structures (~150 per cell [253]) with the length of ~18–20 µm [254] and support the flow of egg liquid, or, after hatching, of water along the skin surface. Besides multiciliated cells, epithelium of larvae skin contains several other non-ciliated cell types: mucus-secreting cells, ionocytes, proton secreting cells, and small secretory cells [255], and thus has a composition which is highly similar to the mammalian airways epithelium (although these epithelia are derived from different germ layers). Cells destined to become multiciliated start to express cilia-specific genes ~12 hpf (stage 11.5), and ciliogenesis starts around stage 20 (~21 hpf). By stage 28 (~32 hpf), assembled cilia are properly oriented [251,256,257].

Motile cilia are also formed in the otic vesicle and by the tadpole floor plate of the spinal cord (monocilia) as well as in brain ventricles (ependymal, mono- and multiciliated cells, forming ~4–9 µm long cilia) 3-4 days after fertilization. They support the circulation of the cerebrospinal fluid [255,258]. Similar to zebrafish, ciliary defects in *Xenopus* embryos can cause laterality defects and hydrocephalus. 

In contrast to mice embryos, in zebrafish or *Xenopus* embryos, cilia are either directly accessible (epidermis, olfactory cilia) or visible because of the embryo’s body transparency (e.g., cilia in KV or pronephros). Thus, the cilia motion can be observed in living embryos. The efficiency of cilia beating can be monitored by tracing the movement of fluorescent beads injected into zebrafish embryo’s KV at 6–10 somite stages or *Xenopus* embryo’s gastrocoel roof plate at stage 17 or into brain ventricles at stage 46 [238,248,258]. Cilia motility in pronephros, spinal canal, olfactory placode (zebrafish) or skin (*Xenopus*), and the estimation of cilia length can also be conducted in living transgenic embryos expressing GFP-tagged ciliary proteins such as Arl13b, or by injection of mRNA encoding GFP-tagged ciliary protein [245,259,260]. 

As mentioned above, the large size of the zygotes and blastomeres of early embryos facilitates the relatively easy introduction of the DNA, mRNA, morpholino, proteins, or drugs into developing embryo, providing convenient tools for the study of cilia assembly and function [233,235,245,260]. Injection of morpholino (MO), a very stable artificial oligonucleotide complementary to mRNA translation start or splice junction, enables efficient gene knockdown. However, the morpholino has to be carefully validated as it may cause toxicity and off-target effects, the issues that can be resolved by the co-injection of mRNA encoded by the targeted gene (rescue experiment) [261]. More recently developed methods involving the transcription activator-like effector nuclease (TALEN) and CRISPR/Cas9 approaches enable gene knockout [262,263]. Particularly noteworthy is the fact that an increasing number of studies has reported the differences in the phenotype of gene morphant and mutant. Bases for such a phenomenon were broadly discussed in a recently published review [264].

Owing to the fact that zebrafish embryos with silenced expression or a mutation of the ciliary genes exhibit characteristic developmental abnormalities, this model organism has been frequently used to investigate the significance of PCD-causative genes. The zebrafish homologs of human PCD-causative genes were listed in a very recent publication [242]. Studies using zebrafish as a model not only reproduced ciliary defects observed in PCD-affected individuals but also helped to understand the molecular basis of such defects. Among PCD-related genes functionally studied in zebrafish embryos are genes encoding (i) factors required for cilia assembly and cell polarity: Foxj1 homologs [265,266], and MCIDAS/multicilin [267], (ii) cytoplasmic proteins required for dynein arm assembly (see Table 1 in [152] for a full list and references), (iii) proteins required for ODA docking: CCDC151 [268], ARMC4 [269], TTC25 [270], CCDC103 [91], (iv) a molecular ruler protein CCDC40 [271], N-DRC subunits CCDC65/DRC2 [111] and GAS8/DRC4 [272,273], radial spoke components RSPH9 [94,274] and NME5 (Nucleoside diphosphate kinase 5) [275], and central pair-associated protein STK36/Fu [276]. 

Interestingly, with the recent use of zebrafish as a model, it was shown that CCDC103, the protein associated with axonemal outer doublets, is also present in myeloid cells where it co-localizes with microtubules. Mutations in *CCDC103* or *SPAG6* reduce microtubule stability and myeloid cell proliferation and migration [277]. Myeloid cells do not assemble cilia [278], but surprisingly, some researchers reported that myeloid cells obtained from PCD-affected individuals have some functional alterations [279,280,281]. 

In comparison to zebrafish, *Xenopus* embryo is a less frequently used model. A vast majority of PCD-causative genes studied in *Xenopus* encoded proteins involved in ciliogenesis and cell polarity control. Those were *FOXJ1* [258,266], *MCIDAS/ Multicilin* [282], CCNO [283,284], and *GAS2L2* [48]. Out of the two studied dynein arms-assembly proteins, ZMYND10 and CFAP298/FBB18/kurly, only kurly morphants assembled multiple cilia with motility defects (additionally, also cell polarity was affected) [285], while *ZMYND10* morphants failed to form cilia despite the presence of numerous centrioles [185]. However, the rescue experiment was not included in this study; thus, it is not clear if the observed phenotype was specific. Moreover, the knockdown of *TTC25* in *Xenopus*, similar to zebrafish, mice, and humans, caused the lack of the outer dynein arms and laterality defects [250,254,270]. Finally, the significance of *CFAP43*, an MMAF-related gene, was also studied in *Xenopus* [286].

Ideally, animal models used to study human diseases should imitate or resemble humans in their genetic and physiologic characteristics. Thus, some types of experiments have to be conducted using mammalian models or in vitro cultured cells or tissues (either ones that are commercially available or derived from cells obtained from PCD-affected individuals). However, even these models have their pros and cons. 

## 5. Mice—Blood Is Thicker than Water—When Being a Mammal Matters

For more than 100 years, the mouse *Mus musculus* has been the most frequently used model organism. It is a truism that among all model organisms used to study PCD-related genes and the impact of their mutations on cilia assembly, structure, and motility, the mouse genome, development, and body organization are the most similar to those of humans [55,287,288,289,290]. Compared to other mammals, the maintenance of this small rodent is comparatively inexpensive, pregnancies are frequent (5–10 times per year), the gestation period is short (~19–20 days), and offspring are relatively numerous (on average 6–8 pups) [287,290,291]. It is also worth adding that mouse multiciliated cells such as mouse tracheal epithelial cells (mTEC) [292,293] or ependymal cells [294,295,296] can be cultured in vitro. 

More importantly, cell biology, biochemistry, and molecular biology methods (both forward and reverse genetic approaches) are remarkably well-developed. The outcome of mutations in PCD-causative genes were studied in mutant mice obtained by (i) N-ethyl-N-nitrosourea mutagenesis (ENU) followed by a forward genetic screen (e.g., *Dnah5* [227,297] *Dnah11* [298,299], *Armc4* [269], *Ccdc39* [227,300], *Ccdc40* [271], *Ccdc151* [268], *Dnaaf1/Lrrc50* [301], and Spef2 [302] ), or reverse genetic approaches, including (ii) knockout mouse generated by traditional gene targeting (gene eliminated in all body cells), e.g., *Dnaaf2/Ktu* [303,304], *Rsph1* [305], and *Dnaaf4/Dyx1c1* [306], or more advanced (iii) conditional knockouts (inactivation of the gene in specific cell types in a certain tissue), e.g., *Spef2* [307,308], or (iv) CRISPR/Cas9 gene editing, e.g.: *Ttc25* [254], *Zmynd10/ Dnaaf7* [189], *Dnajb13* [309], and *Mcidas* [310]. It should also be noted that mouse lines facilitating further genetic manipulations and the analysis of ciliated cells have recently been generated [311,312]. Finally, genetic manipulations of PCD-causative genes and their outcome can be analyzed in vitro using mouse tracheal epithelial cells (mTECs) [282]. 

The activity and/or structure of cilia in MCCs can be evaluated in vivo, ex vivo, or in vitro using radioactive particles, fluorescent beads, or lead dust and different, sophisticated microscopic systems [17,227,313,314,315,316,317,318].

Although motile cilia are present in mice and humans in the same organs, the phenotypic outcome of the mutation of PCD-causative genes is not identical. Mice carrying a mutation in ciliary genes frequently develop hydrocephalus, while in PCD-affected patients, such a condition is very rare. This striking discrepancy is likely due to anatomical differences between murine and human brain ventricles and additional specific genetic modifiers that segregate in inbred mouse strains, as was earlier suggested [287,319,320,321]. 

Without a doubt, over the years, research conducted using the mouse model has brought significant progress regarding genes and molecular mechanisms underlying PCD and motile ciliary function. These data were brought together recently in a splendid review article [287]. Therefore, we omit discussing this aspect here. 

## 6. In Vitro Cell Culture 

Assessment of cilia motion and ultrastructure are important tools in PCD diagnosis. However, the quality of samples obtained during biopsies is often poor due to secondary changes caused by infections or sampling itself. In consequence, the diagnosis can be challenging [322]. Moreover, the amount of the biological material obtained from the patient may not be sufficient to conduct all currently available tests. The culturing of cells collected during the nasal brushing or biopsy can overcome these problems and improve the diagnosis. More importantly, such cell culture “models” are patient-specific [323,324,325].

The airway epithelium is composed of several cell types, including progenitor basal cells, club cells, goblet cells, and multiciliated cells (for details, see [47]). Ciliated cells account for 50–70% of the epithelial cell population [326]. Basal cells bear an important feature - during asymmetric division, one of daughter cells maintains stem cell’s properties while the other one differentiates. Basal cells bear an important feature—during asymmetric division, one of the daughter cells maintains stem cell properties while the other one differentiates. 

In vitro, cell differentiation and ciliation take place when cultured cells are attached to the floating collagen, form floating spheroids, or are grown under conditions that expose the apical part of the cell culture to air (air–liquid interface, ALI) [323,325,327,328,329,330]. A full differentiation of such cultured airway epithelium takes approximately 3–4 weeks [331]. Differentiated cultures can be maintained for more than several weeks or even months. 

Obviously, cultures of cells derived from affected individuals and differentiating into airway-like epithelium are the most adequate tool for accurate understanding ciliary defects in PCD patients. However, in comparison with other PCD models, airway epithelial cell cultures are significantly more expensive and, because of limited availability of material, can be used only in several test types (e.g., light and electron microscopy, low-cell-number or single-cell analyses). 

Up to now, ALI cultures have been used to support the analysis of PCD-causing mutations, including the mutation in *CFAP300/C11orf70* [221], *CCDC65* [332], *DNAH9* [333], *DNAH5* [334], *HYDIN*, [335], and *FOXJ1* [336]. 

The determination of conditions that enable the reprogramming of somatic cells into induced pluripotent stem cells (iPSC) (for review, see [337]) and the induction of the iPSC differentiation into mature multiciliated cells [338] are the next significant step forward in PCD-related research. In fact, several research groups have reported the establishment of human iPSCs (hiPSCs) from somatic cells of patients with primary ciliary dyskinesia carrying mutations in *CCDC40* [339], *DNAH5* [340], *NME5* [341], *CCNO* [342,343]. Another research group moved a step further and showed that iPSCs can be differentiated into airway basal cells, resembling those in the airway epithelium [344,345]. 

More importantly, the generation of patient-derived iPSCs and their differentiation into airways epithelium, together with genome editing technologies, represent a major tool for developing personalized PCD therapies in future.

## 7. Conclusions

Without a doubt, the research conducted using unicellular and multicellular model organisms has brought about enormous progress in our understanding of molecular, proteomic, and ultrastructural bases of cilia assembly and functioning, and has thus helped to elucidate ciliary abnormalities in PCD-affected individuals, and to consider novel genes as putative PCD-causative. Moreover, studies in the vertebrate models have shed light on some aspects of tissue and organ development. However, even such a closely related model as that of the mouse is not ideal for studying all aspects of PCD and successfully developing appropriate therapies. The situation is even more complicated as PCD is a heterogeneous disorder, and some PCD patients have more acute symptoms, while others have milder ones, depending on the mutated gene or even the type and position of the mutation in the same gene. On the other hand, the entire genetic background of the affected individual may also matter. The recent progress in the methods of hiPSC culture and their reprogramming give hope for the development of personalized therapies and drug testing. 

## Data Availability

Not applicable.
